# Synthesis of Ultrathin WS_2_ Nanosheets and Their Tribological Properties as Lubricant Additives

**DOI:** 10.1186/s11671-016-1659-3

**Published:** 2016-10-01

**Authors:** Xianghua Zhang, Hongxiang Xu, Jiangtao Wang, Xia Ye, Weining Lei, Maoquan Xue, Hua Tang, Changsheng Li

**Affiliations:** 1 School of Mechanical Engineering, Jiangsu University of Technology, Changzhou, 213001 Jiangsu Province China; 2 School of Materials and Engineering, Jiangsu University of Technology, Changzhou, 213001 Jiangsu Province China; 3 Changzhou Vocational Institute of Light Industry, Changzhou, 213164 Jiangsu Province China; 4 School of Materials Science and Engineering, Jiangsu University, Zhenjiang, 212013 Jiangsu Province China

**Keywords:** WS_2_, Nanosheets, Lubricant additives, Tribological properties

## Abstract

In this paper, ultrathin WS_2_ nanosheets with thickness of about 5 nm were successfully prepared by a facile solid phase reaction method. The as-synthesized samples were characterized by X-ray diffraction (XRD), scanning electron microscopy (SEM), and transmission electron microscopy (TEM). On the basis of experimental results obtained under different reaction durations, a possible formation mechanism of WS_2_ nanosheets is proposed. The tribological performance of ultrathin WS_2_ nanosheets as additives in the 500SN base oil was tested by an UMT-2 ball-on-disc tribotester, and the worn surface of the steel disc was investigated by a non-contact optical profile testing instrument and SEM. The results showed that the friction coefficient and anti-wear property of base oil can be improved strikingly by adding ultrathin WS_2_ nanosheets. Especially, when the concentration of WS_2_ nanosheets was 1.0 wt.%, the corresponding lubricating oil exhibited the best tribological properties. Moreover, according to the investigation of the wear scar, an anti-friction and anti-wear mechanism is proposed. It is believed that the reduction of friction and wear must come from the addition of ultrathin WS_2_ nanosheets which can penetrate and enter the friction interface and form a continuous tribofilm on the rubbing face.

## Background

Inspired by the discovery of graphene [1] in 2004, ultrathin two-dimensional (2D) nanomaterials have aroused tremendous research interest in recent years. Due to the surface effects associated with their ultrathin structure, ultrathin 2D nanomaterials present many unusual properties [2–6]. For example, graphene [7, 8], a single-layer 2D carbon material, presents outstanding electronic, mechanical, and thermal properties [9, 10]. Besides, other ultrathin 2D nanomaterials, such as transition metal dichalcogenides [11], transition metal oxides [12], and hexagonal boron nitride [13], have also been extensively researched and applied in catalysis, energy storage, and electronics.

Recently, the tribology properties of ultrathin 2D nanomaterials also attracted great attention from researchers. Zhang et al. [14] reported that graphene effectively improved the tribological properties of the lubricant oils. Fan et al. [15, 16] researched the tribological performances of graphene oxide and graphene as multialkylated cyclopentane additives and found that the excellent tribological properties are attributed to the formation of a graphene-rich tribofilm on the sliding surfaces. Chen et al. [17] demonstrated that the ultrathin structure of the MoS_2_ nanosheets makes them very easy to enter the contact area and prevent direct contact of the tribopairs. WS_2_, a representative layered 2D nanomaterial, is also an excellent lubricant due to the easy interlayer sliding. Over the past few decades, WS_2_ nanoparticles with various morphologies and sizes have been synthesized and they are proved to be good lubricant additives [18–22]. However, the friction and wear behavior of ultrathin WS_2_ nanosheets has not previously been investigated.

Until now, many approaches have been used to prepare ultrathin WS_2_ nanosheets, and the top-down and bottom-up strategies are two major synthesis routes. The top-down methods rely on the exfoliation of layered bulk crystals, which include the mechanical cleavage and chemical exfoliation [23–26]. However, these approaches are frequently deficient in the yield and the manufacturing processes of these approaches are complicated. Recently, the bottom-up approaches have been used to synthesize few-layered WS_2_ nanosheets. For example, Yang [27] and Ratha et al. [28] synthesized the ultrathin WS_2_ nanosheets by a one-pot hydrothermal reaction of WCl_6_ and thioacetamide at 265 °C for 24 h. But, it still remains a major challenge to develop simple, reproducible, and economical synthetic approach for the fabrication of ultrathin WS_2_ nanosheets.

In this study, the well-dispersed ultrathin WS_2_ nanosheets have been prepared through a facile solid phase reaction method using WO_3_ and thiourea as raw materials. Tribological performances of WS_2_ nanosheets as additives in the 500SN base oil were also examined using a tribotester. Moreover, the anti-wear and anti-friction mechanisms of WS_2_ nanosheets have also been analyzed.

## Methods

### Synthesis of Ultrathin WS_2_ Nanosheets

All chemical reagents were of analytical grade and applied directly without further purification. Tungsten trioxide (WO_3_) and thiourea were purchased from Aladdin Chemical Reagent Company. A typical synthesis process was carried out as follows: Firstly, 1 mmol tungsten trioxide and 60 mmol thiourea were mixed in an agate mortar and milled by a pestle for 30 min, and then the milled mixture was loaded into a corundum crucible. The temperature of a tube furnace was raised to 850 °C at a rate of 10 °C/min, and the crucible was quickly pushed into the hot zone of the tubular furnace and heated at 850 °C for 1 h under nitrogen atmosphere. After 1 h, the sample was cooled to room temperature and the black sample was collected. The calculated yield was about 86.3 % on the basis of W.

### Characterization

The phase composition of the as-prepared sample was investigated by a Japan Shimadzu LabX XRD-6000 X-ray diffractometer equipped with Cu Kα radiation (λ = 0.1546 nm). The morphologies and sizes of the as-synthesized products were observed by a field scanning electron microscope (FESEM; JEOL JSM-7001 F) equipped with energy-dispersive spectrum (EDS) and a transmission electron microscope (TEM; JEM-2100).

### Tribological Property Test

Tribological properties were tested by a UMT-2 multi-functional tribotester (CETR, USA). The as-synthesized WS_2_ powders modified by dispersing agent sorbitol monooleate (Span-80) were dispersed into the 500SN base oil by ultrasonication for 1 h, and then a series of samples with different contents of WS_2_ were acquired. The friction and wear tests were conducted using a ball-on-disc mode with applied load of 10–60 N and rotating speed of 50–400 rpm for 30 min. The fixed upper specimen (ball) was made of GCr15 bearing steel (AISI 52100) with a diameter of 10 mm and a hardness of 62 HRC. The rotating lower specimen (disc) was made of 45# steel with a diameter of Φ40 mm and a thickness of 3 mm. Each test was repeated three times in order to minimize data scatter. The friction coefficient was recorded automatically by the tribometer, and the wear scar diameters were tested by a PS50 non-contact optical profile testing instrument (NANOVEA Inc., USA). Morphologies of wear scars were investigated by SEM. And EDS spectroscopy was used to analyze the elements of the friction surface. The X-ray photoelectron spectroscopy (XPS) of the chemical states of some typical elements on the worn surfaces was recorded with a PHI5702 X-ray photoelectron spectrometer.

## Results and Discussion

### Structural and Morphological Characterization of Ultrathin WS_2_ Nanosheets

**Fig. 1:**
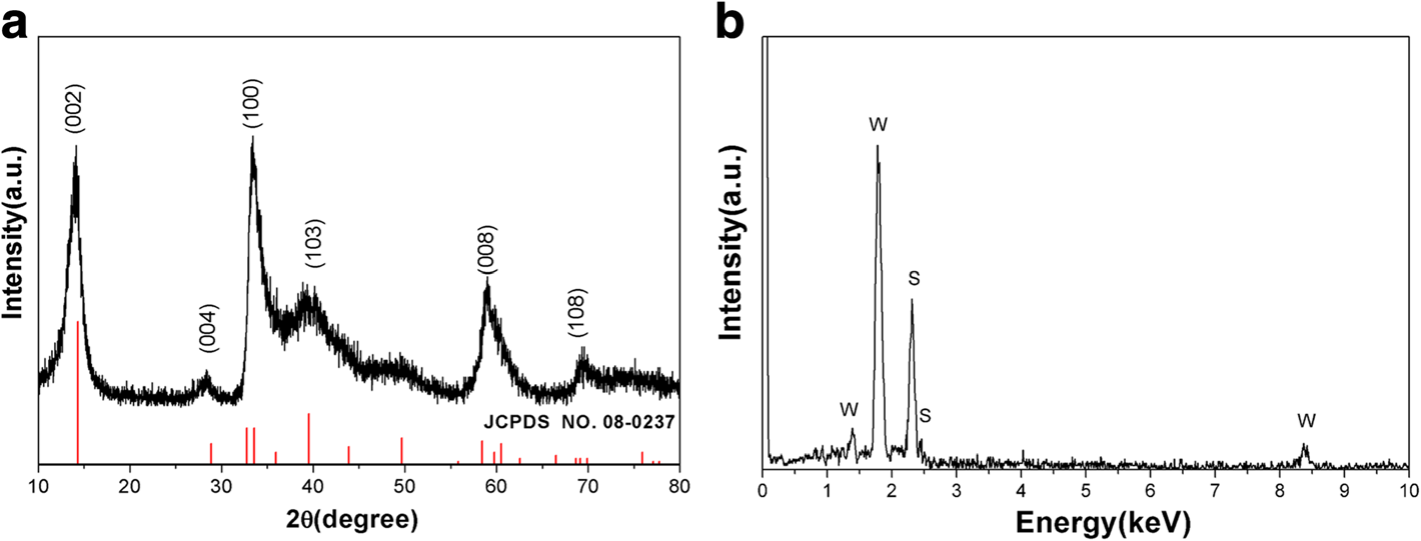
**a** XRD pattern and **b** EDS of the as-prepared WS_2_ nanosheets

The crystalline structure and phase composition of the synthesized samples were confirmed by powder X-ray diffraction. A typical XRD pattern of the obtained ultrathin WS_2_ nanosheets is shown in Fig. 1a. It can be observed that all the reflection peaks can be perfectly indexed to the hexagonal WS_2_ phase with cell constants of a = 3.154 Å and c = 12.362 Å, which is consistent with the standard JCPDS card no. 08-0237. No peaks of other impurity phases are detected from this pattern. EDS spectrum is presented in Fig. 1b, which reveals that the sample consisted of elements W and S; no other element is observed. Moreover, the quantification of the peaks shows that the molar ratio of W to S is about 1:1.95, which is almost consistent with the stoichiometric WS_2_.

**Fig. 2:**
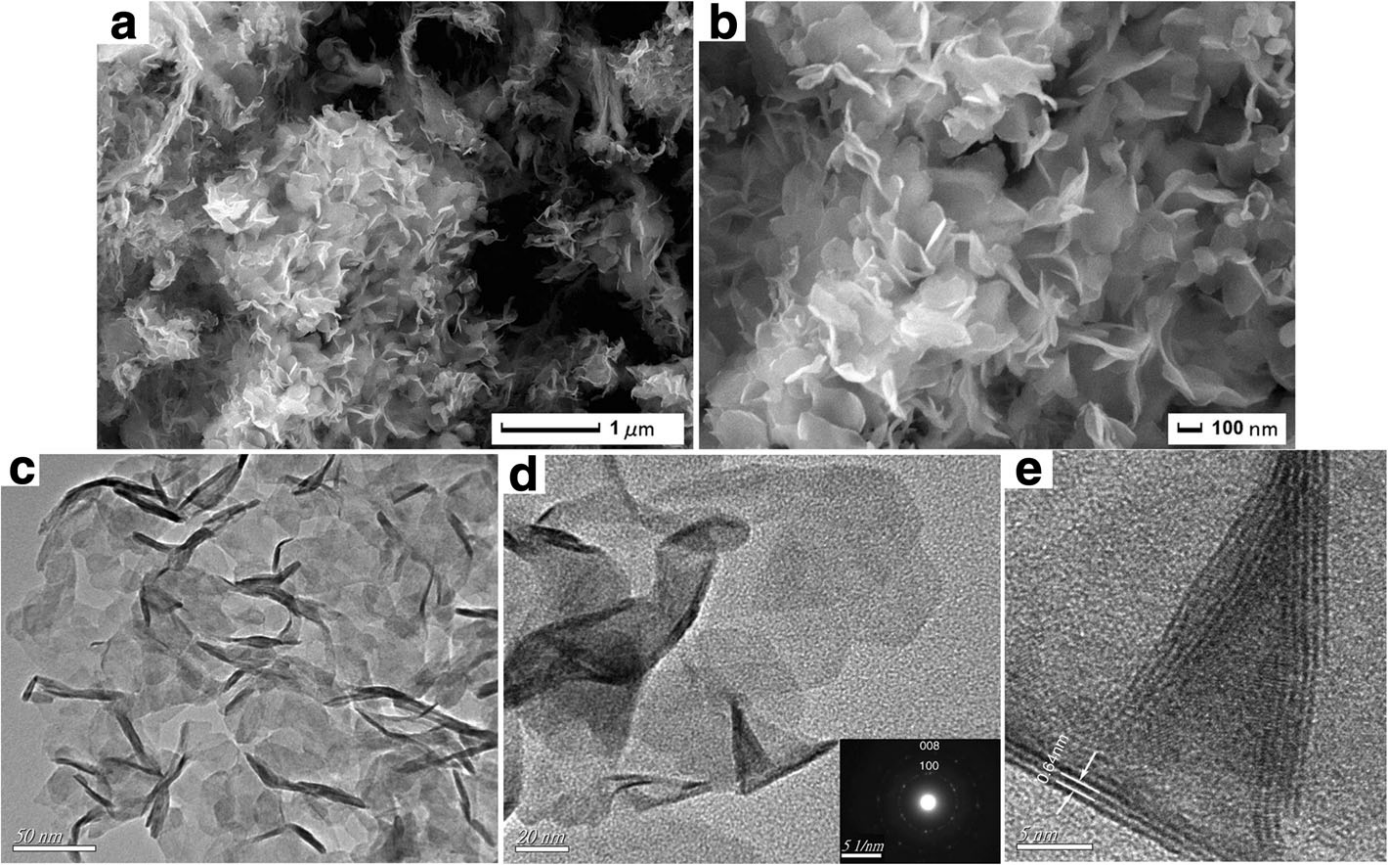
FESEM (**a**, **b**) and TEM (**c**, **d**, **e**) images of the WS_2_ nanosheets obtained at 850 °C for 1 h

The morphology and microstructure of the as-prepared samples were investigated by FESEM and TEM. The typical FESEM images of the obtained samples at different magnifications were showed in Fig. 2a, b. As shown in Fig. 2a, the low-magnification SEM image, the prepared WS_2_ samples were mainly made up of a large number of nanosheets, which are similar to the morphology reported by Wu et al. [29, 30]. To further expose the microstructure of these sheets, the high-magnification SEM image (Fig. 2b) is also observed. It shows that the prepared nanosheets are nearly monodisperse and stacked loosely, and the width of the nanosheets is in the range of 100–200 nm; the thickness of the nanosheets is ~5 nm. The size is much thinner than the reported nanoplates in literatures [31, 32]. Figure 2c–e shows TEM images of the as-synthesized samples. The low-magnification TEM image (Fig. 2c) clearly shows that the as-prepared samples are predominantly comprised of ultrathin and well-dispersed sheets. And the TEM image is in accordance with the morphology as presented in the SEM pictures. Moreover, similar to the reported by Wu [29, 30], it was also observed that a fraction of WS_2_ nanosheets were slighted curved. The reason maybe that such ultrathin 2D materials are unstable and easy to form closed structures by rolling up owing to eliminate of dangling bonds at the edges [30, 33]. In Fig. 2d, the structure of ultrathin sheets is presented more clearly. The selected area electron diffraction (SAED) pattern for the WS_2_ sheets (inset in Fig. 2d) shows two dotted diffraction rings. The diffraction rings are in accordance with the reflections of the tungsten sulfide (100) and (008) planes. The high-magnification TEM image, as shown in Fig. 2e, indicates that the interlayer distance between the WS_2_ layers is about 0.64 nm, which corresponds to the (002) plane of WS_2_.

**Fig. 3:**
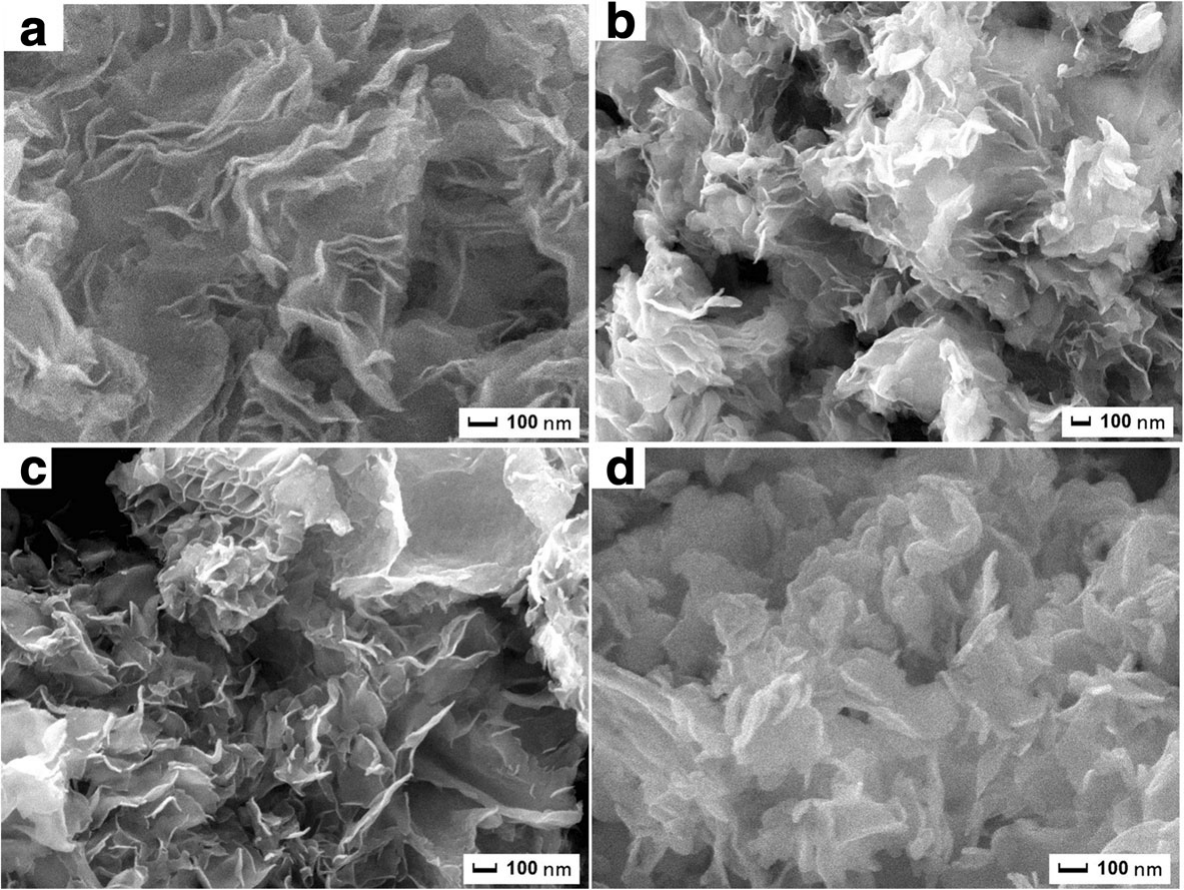
FESEM images of the sample for **a** 10 min, **b** 30 min, **c** 2 h, and **d** 4 h

In order to understand the formation process of these ultrathin nanosheets, we carried out different reaction duration (10 min, 30 min, 2 h, and 4 h) experiments and compared the morphologies of the samples harvested at different stages. Figure 3a–d shows their FESEM images. From Fig. 3a, we can find that a few nanosheets are observed after a reaction time of 10 min. But these nanosheets interconnected with each other and the sample presents an etched trace. When the reaction time is 30 min, the nanosheets were becoming dispersed, showing a significant lamellar structure (Fig. 3b). Further prolonging the reaction time to 2 h, the size of the nanosheet becomes large; dispersion becomes well (Fig. 3c). When the reaction time is extended to 4 h, the thickness of nanosheets significantly increased (Fig. 3d). These results illustrated that the reaction time is conducive to the growth of nanosheet, but the longer reaction time will lead to the thickness increasing.

To date, many literatures [21, 22, 29, 30, 33] have reported synthesis of different WS_2_ nanostructures by high-temperature sulfurization reaction and discussed the formation mechanism of these nanomaterials. For example, Tenne [33, 34] synthesized fullerene-like WS_2_ nanomaterials using WO_3_ and H_2_S as reactants at 850 °C and proposed the outside-in sulfurization mechanism. Wu et al. [29, 30] prepared the WS_2_ nanosheets using WO_3_ and S as raw materials at 600 °C and put forward a reaction mechanism of mechanical activation and exfoliation. Based on the above experimental results, we deduce that the formation mechanism of WS_2_ nanosheets prepared by our approach is different from the above reports. The formation process maybe includes a two-step reaction, similar to the formation mechanism of MoS_2_ nanosheets which is reported in the literatures [35]. When WO_3_ and thiourea were pushed into the tubular furnace at 850 °C, thiourea rapidly decomposed and produced large amounts of CS_2_ [36]. And the released CS_2_ reacted with WO_3_ and formed a WS_2_ layer on the surface of WO_3_ particles. On the other hand, thiourea decomposition will release a great deal of NH_3_ and H_2_NCN, and these released gases will produce corrosive effects on the particles surface and exfoliated the WS_2_ layer. So, when the reaction time is short, the exfoliating process is not complete and the obtained samples present an interconnected structure. The reaction process in our experiment could be expressed as follows:

1 $$ 2\mathrm{S}\mathrm{C}{\left(\mathrm{N}{\mathrm{H}}_2\right)}_2\to \mathrm{C}{\mathrm{S}}_2+{\mathrm{H}}_2\mathrm{N}\mathrm{C}\mathrm{N}+2\mathrm{N}{\mathrm{H}}_3 $$

2 $$ 3\mathrm{C}{\mathrm{S}}_2+2\mathrm{W}{\mathrm{O}}_3\to 2\mathrm{W}{\mathrm{S}}_2+3\mathrm{C}{\mathrm{O}}_2+2\mathrm{S} $$

### Friction and Wear Property Analysis

**Fig. 4:**
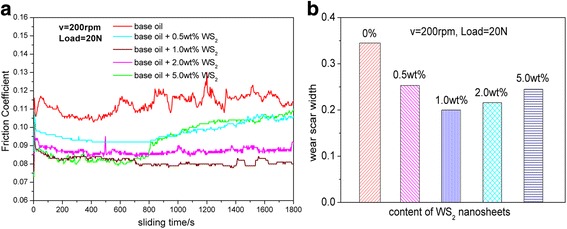
**a** Friction coefficient as a function of sliding time and **b** wear scar width on disc specimens lubricated with different concentrations WS_2_ nanosheets in 500SN base oil

The tribological properties of 500SN oil and base oil with the as-synthesized WS_2_ nanosheets were measured by the UMT-2 tribotester. The influences of WS_2_ nanosheet concentration on the friction coefficient and wear scar diameters under 20-N load with rotating speed of 200 rpm are presented in Fig. 4. Figure 4a depicts the real-time friction coefficients of the tribopairs lubricated by 500SN base oil with adding different amount of the WS_2_ nanosheets. It can be noted that the curve of real-time friction coefficient of pure base oil is unstable. When ultrathin WS_2_ nanosheet is added into the base oil, friction coefficient drops obviously and the friction coefficient curve turns smooth. Especially, the friction coefficient has a largest decrease and presents more stable when the WS_2_ nanosheet concentration is 1.0 wt.%. It is also observed that the real-time friction coefficient decreases first and then have a light increased when the WS_2_ nanosheet concentration is 0.5 or 5.0 wt.%.

The wear scar diameters (WSD) of the steel disc that is lubricated by different oil samples are shown in Fig. 4b. It demonstrates that the wear scar diameters are obviously decreased with adding WS_2_ nanosheets. As the additive concentration is 0 %, the WSD is close to 0.35 mm. With increasing the additive concentration to 0.5 wt.%, the WSD decreases to 0.25 mm. When WS_2_ nanosheet concentration increases to 1.0 wt.%, the WSD is smallest and is about 0.2 mm, which is nearly 43 % reduction for the WSD of the pure base oil. But, if the additive concentration is further increased to 2.0 or 5.0 wt.%, the WSD presents a larger trend. Therefore, we can conclude that there is an optimum concentration for WS_2_ corresponding to 1.0 wt.% for their anti-wear abilities. At the same time, all of these results demonstrate that the ultrathin WS_2_ nanosheets can greatly improve the anti-friction and anti-wear properties of 500SN oil.

**Fig. 5:**
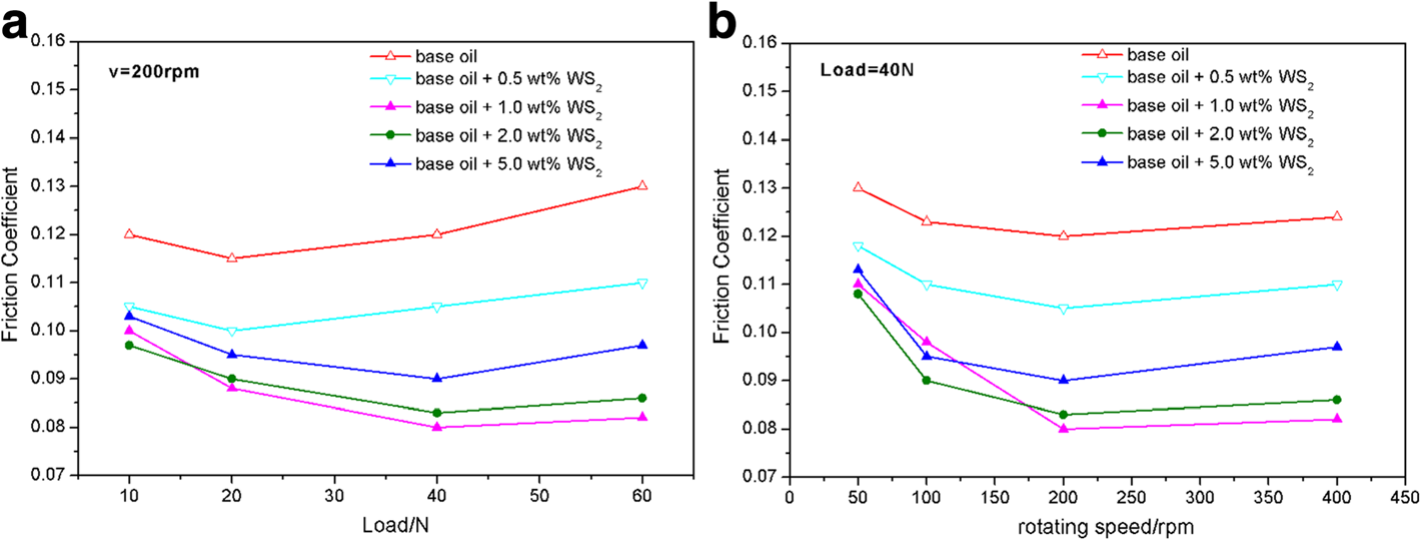
Variation of average friction coefficient with load at a constant rotating speed of 200 rpm (**a**) and with rotating speed at a constant load of 40 N (**b**)

In order to further evaluate the anti-friction and anti-wear properties of ultrathin WS_2_ nanosheets, a series of compared experiments were conducted by changing the working load and rotating speed. Figure 5a shows the variation of the average friction coefficients with applied loads at a constant rotating speed of 200 rpm for 30 min. It is also found that the friction coefficients of base oil with WS_2_ nanosheets are all lower than pure base oil at different loads. When the applied load increases from 10 to 40 N, the average friction coefficients have a decreasing tendency. But when the load increased to 60 N, the average friction coefficients have a little increase. Especially, when the WS_2_ nanosheet concentration is 1.0 or 2.0 wt.%, the average friction coefficients are lower and stable than other mass fraction.

Figure 5b shows the variation of the average friction coefficient with rotating speeds for all the oil samples. When the rotating speed is 50 rpm, all the average friction coefficients of the five kinds of oil samples are high. The friction coefficient is observed to decrease with increasing rotating speed for all the lubricating oil.

**Fig. 6:**
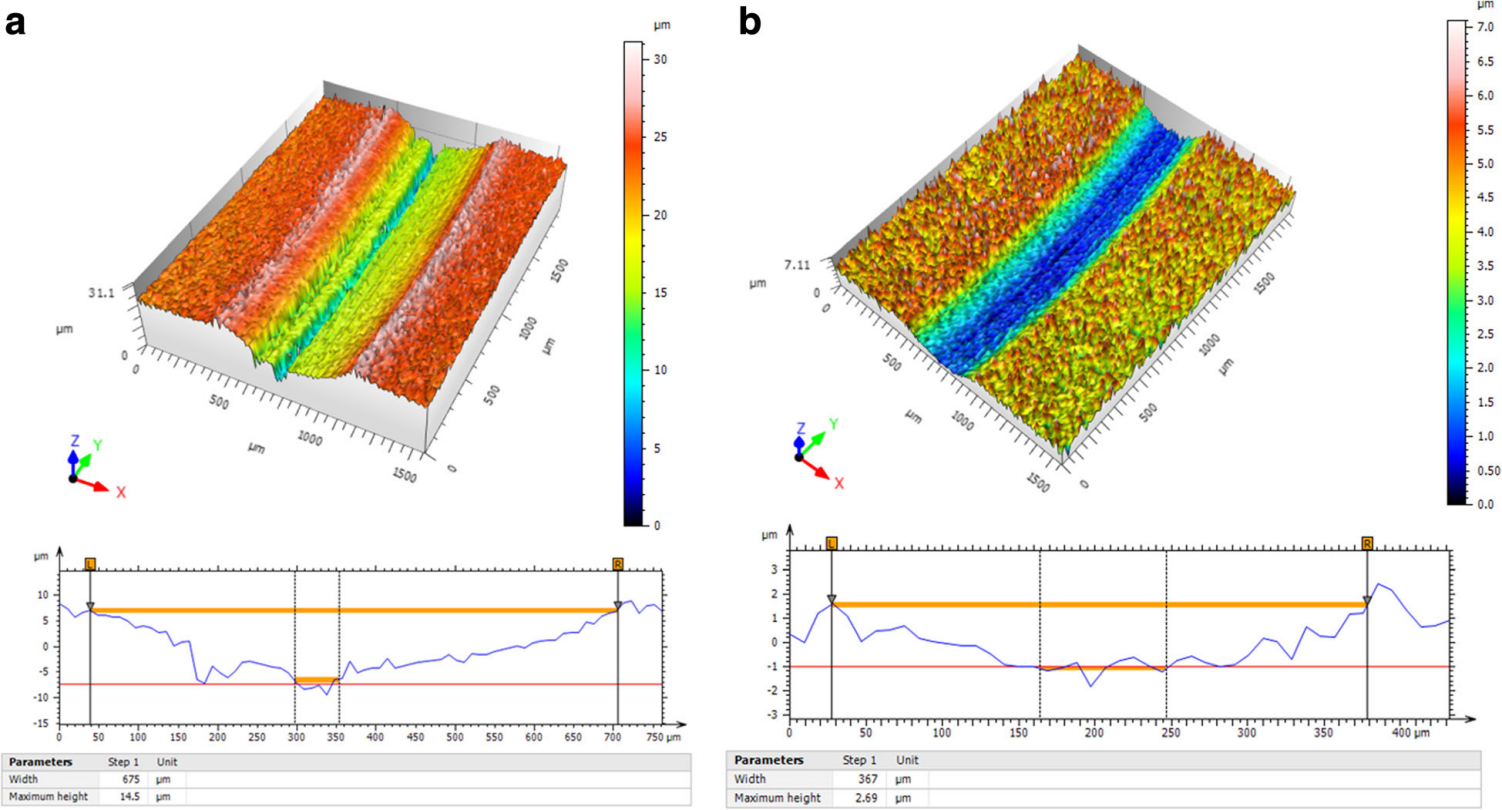
Non-contact optical profile testing instrument images of wear scars at 200 rpm under 40 N for 30 min. **a** For base oil. **b** For base oil with 1.0 wt.% ultrathin WS_2_ nanosheet

To further analyze the anti-wear performance of WS_2_ nanosheets, a PS50 non-contact optical profile testing instrument was used to investigate the wear surface morphology. Figure 6 presents the three-dimensional (3D) surface morphologies of the wear track on the steel disc after running for 30 min under 40 N in base oil and in base oil with 1.0 wt.% WS_2_ nanosheets. As shown in Fig. 6, it can be clearly seen that the depth and width of the wear track for base oil with 1.0 wt.% WS_2_ nanosheets are about 2.69 and 367 μm (Fig. 6b), while those for pure base oil are about 14.5 and 675 μm (Fig. 6a). Moreover, the rubbing surface shown in Fig. 6a is quite rough and many wide and deep furrows and grooves distribute on the surface. On the contrary, the rubbing surface shown in Fig. 6b is smoother. This also verifies that the ultrathin WS_2_ nanosheets play a significant role in improving anti-wear ability of base oil.

**Fig. 7:**
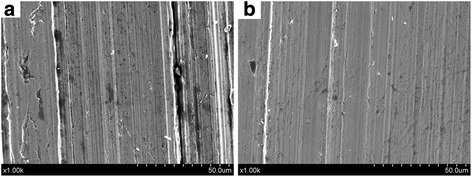
SEM images of the worn surfaces with load of 40 N at 200 rpm for 30 min lubricated by **a** pure base oil and **b** base oil with 1.0 wt.% WS_2_ nanosheets

In order to inspect the anti-friction and anti-wear mechanisms of WS_2_ nanosheets, we used SEM and EDS to analyze the worn surface. Figure 7 presents the SEM images of the worn surface of steel disc which lubricated by base oil and the base oil with 1.0 wt.% WS_2_ nanosheets at 40 N for 30 min. From Fig. 7a, it can be noted that the worn surface of lubricated by pure base oil presents many wide and deep furrows, which is consistent with the morphology as presented in Fig. 6. It proves that the surface suffers very serious wear during the friction process. On the other hand, the worn surface of lubricated by base oil with 1.0 wt.% WS_2_ nanosheets is smooth and the scratches on the worn surfaces are slighter and more uniform (Fig. 7b). This further demonstrates that ultrathin WS_2_ nanosheets have good anti-wear property.

**Fig. 8:**
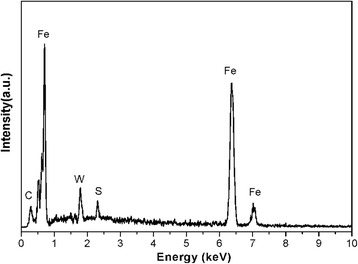
EDS of the worn surface of the steel disc lubricated by base oil with 1.0 wt.% WS_2_ nanosheets

**Fig. 9:**
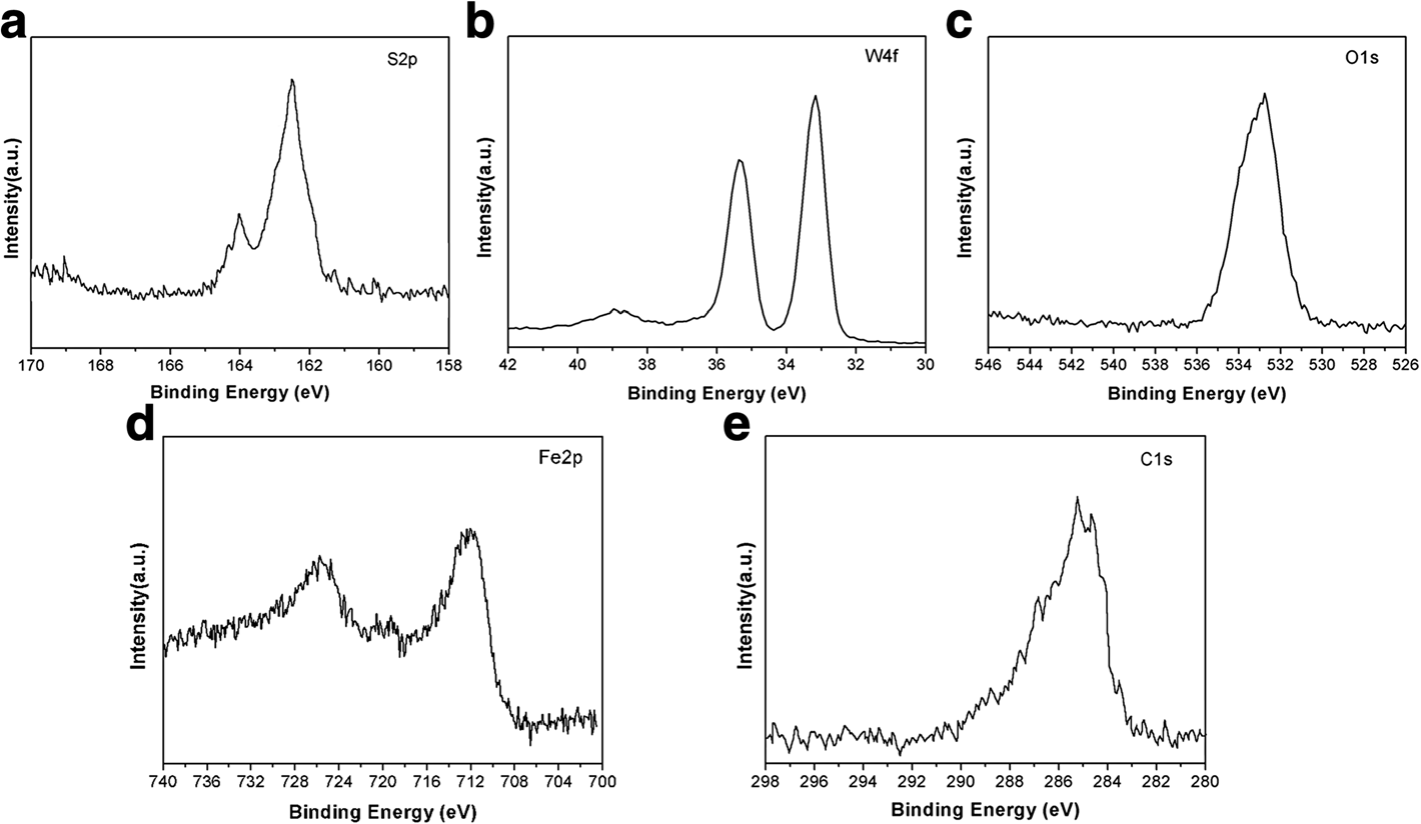
XPS spectra of **a** S 2p, **b** W 4f, **c** O 1 s, **d** Fe 2p, and **e** C 1 s on the worn surfaces lubricated by base oil with 1.0 wt.% WS_2_ nanosheets

Figure 8 depicts the EDS spectra obtained from the worn surface of the steel disc lubricated by base oil with 1.0 wt.% WS_2_ nanosheets at 40 N for 30 min. It can be noted that the element of W and S is detected from the worn surface. To further investigate the chemical state of elements in the worn surface, the wear tracks were analyzed by XPS. The typical XPS analysis results are shown in Fig. 9, which reveals the XPS spectra of S 2p, W 4f, O 1 s, Fe 2p, and C 1 s. The S 2p peak was detected at 162.4 eV, and two W 4f peaks were detected at 32.8 and 35.1 eV, which is attributed to the presence of WS_2_ on the worn surface [37, 38]. The O 1 s peak appearing at around 532.5 eV and the Fe 2p peaks at 710.8 indicate that Fe_2_O_3_ appears on the worn surfaces. The C 1 s peak at 284.8 eV reveals the existence of C–O [39].

### Anti-friction and Anti-wear Mechanism Discussion

The anti-friction and anti-wear mechanisms of nanomaterials as lubricant additives have been discussed in many literatures, which can be summarized as the following three reasons: the first one is the rolling effect [19, 20], the second reason is the tribofilm or tribolayer effect [38, 40–42], and the last one is the surface mending effect [43, 44]. Based on the above experiment results, the mechanism of WS_2_ nanosheets reducing friction and wear can be attributed to the formation of tribofilm on the rubbing face. When WS_2_ is used as a lubrication additive, WS_2_ nanosheets will be easy to penetrate and enter the interface of the rubbing face and be adsorbed on the surface of the tribopairs. Then, the adsorbed WS_2_ will form a continuous tribofilm on the rubbing face and the tribofilm can improve the tribological properties of the base oil by decreasing the shearing stress and yielding low wears. Moreover, the tribofilm is able to endure high load than pure base oil. So, we can conclude that the main reason of WS_2_ nanosheets reducing friction and wear is based on the two-dimensional sheet shape, which reduces the friction between the tribopair by the shearing of layers and forming tribofilm on the rubbing surface [45].

The effect of the WS_2_ nanosheet additive concentration and rotating speed on the tribology properties can be explained as follows. When the concentration of the additive is low, e.g., 0.5 wt.%, there is not enough nanosheets to form a continuous film. On the contrary, if the concentration of WS_2_ nanosheets is too high, more nanosheets would aggregate and chemical condensation would appear [46]. This would aggravate the friction and wear. Therefore, there is an optimal concentration of nanosheets as lubricant additives, which is 1.0 wt.% for WS_2_ nanosheets. The reason that the friction coefficient decreased with the rotating speed increasing can be attributed to the fact that with the increase of sliding velocity, the shear stress increased, which is beneficial for the formation of tribofilm. Hence, a decreased friction coefficient was reached [47].

## Conclusions

In summary, ultrathin WS_2_ nanosheets with thickness of ~5 nm were successfully prepared by a solid phase reaction. The anti-friction and anti-wear performance of base oil can be significantly improved by adding the ultrathin WS_2_ nanosheets, and the optimum nanosheet concentration is 1.0 wt.%. Moreover, the experiment results indicated that WS_2_ nanosheets penetrate into the friction interface and form a continuous tribofilm on the rubbing face, which could improve the anti-friction anti-wear properties. So, WS_2_ nanosheets reveal a large potential in lubrication.

## Abbreviations

EDS: Energy-dispersive spectrum
FESEM: Field scanning electron microscope
SAED: Selected area electron diffraction
SEM: Scanning electron microscopy
TEM: Transmission electron microscopy
WSD: Wear scar diameters
XPS: X-ray photoelectron spectroscopy
XRD: X-ray diffraction
